# Mesenchymal Stem Cells May Ameliorate Nephrotic Syndrome Post-Allogeneic Hematopoietic Stem Cell Transplantation-Case Report

**DOI:** 10.3389/fimmu.2017.00962

**Published:** 2017-08-14

**Authors:** Xin Zhang, Yanwen Peng, Zhiping Fan, Ke Zhao, Xiaoyong Chen, Ren Lin, Jing Sun, Guobao Wang, AndyPeng Xiang, Qifa Liu

**Affiliations:** ^1^Department of Hematology, Nanfang Hospital, Southern Medical University, Guangzhou, China; ^2^Third Affiliated Hospital of Sun Yat-sen University, Guangzhou, China; ^3^Center for Stem Cell Biology and Tissue Engineering, The Key Laboratory for Stem Cells and Tissue Engineering, Ministry of Education, Sun Yat-Sen University, Guangzhou, China; ^4^Department of Nephrology, Nanfang Hospital, Southern Medical University, Guangzhou, China; ^5^Department of Biochemistry, Zhongshan Medical School, Sun Yat-Sen University, Guangzhou, China

**Keywords:** mesenchymal stem cells, allogeneic hematopoietic stem cell transplantation, nephrotic syndrome, chronic graft-versus-host disease, regulatory B cell

## Abstract

**Introduction:**

Because of their immunomodulatory and anti-inflammatory effects, mesenchymal stem cells (MSCs) have been considered as potential therapeutic agents for treating immune-related or autoimmune diseases, such as graft-versus-host disease (GVHD). Nephrotic syndrome (NS) after allogeneic hematopoietic stem cell transplantation (allo-HSCT) is an uncommon complication with unclear etiology and pathogenesis. It may be an immune disorder involving immune complex deposition, B cells, regulatory T cells (Tregs), and Th1 cytokines and be a manifestation of chronic GVHD. Corticosteroids and calcium antagonists, alone or in combination, are the most common therapeutic agents in this setting. Rituximab is commonly administered as salvage treatment. However, treatment failure and progressive renal function deterioration has been reported to occur in approximately 20% of patients in a particular cohort.

**Case presentation:**

We present a patient who developed NS 10 months after allo-HSCT. After treatment failure with cyclosporine A, prednisone, and rituximab, she achieved a complete response with MSC treatment. The clinical improvement of this patient was accompanied by a decreased B cell population together with an increased frequency of regulatory B cells (Bregs) and Tregs after MSC treatment.

**Conclusion:**

MSCs could modulate NS after allo-HSCT by suppressing B cell proliferation, inducing Tregs and Bregs, and inhibiting inflammatory cytokine production by monocytes and NK cells. Among all these, Bregs might play an important role in ameliorating the NS of this patient.

## Introduction

Mesenchymal stem cells (MSCs) are multipotent stem cells that can be isolated from various adult tissues including umbilical cord blood, adipose tissue, muscle, and dental pulp. There has been a focus on the immunomodulatory and anti-inflammatory effects of MSCs as potential therapeutic agents for treating immune-related or autoimmune diseases, such as graft-versus-host disease (GVHD) (1). Nephrotic syndrome (NS) after allogeneic hematopoietic stem cell transplantation (allo-HSCT) with unclear etiology and pathogenesis. We and others have suggested that NS after allo-HSCT might be a manifestation of chronic GVHD (cGVHD) and occurs in isolation or accompany other manifestations of cGVHD (2–4). Corticosteroids and calcium antagonists [cyclosporine A (CsA) or tacrolimus], alone or in combination, are the most common therapeutic agents in this setting. Rituximab is commonly administered as salvage treatment. However, treatment failure and progressive renal function deterioration has been reported to occur in approximately 20% of patients in a particular cohort (4). In this case report, we present a patient with NS after allo-HSCT who was unresponsive to CsA and prednisone, poorly responsive to rituximab, achieved a complete response with MSC treatment.

## Case Report

**Figure 1:**
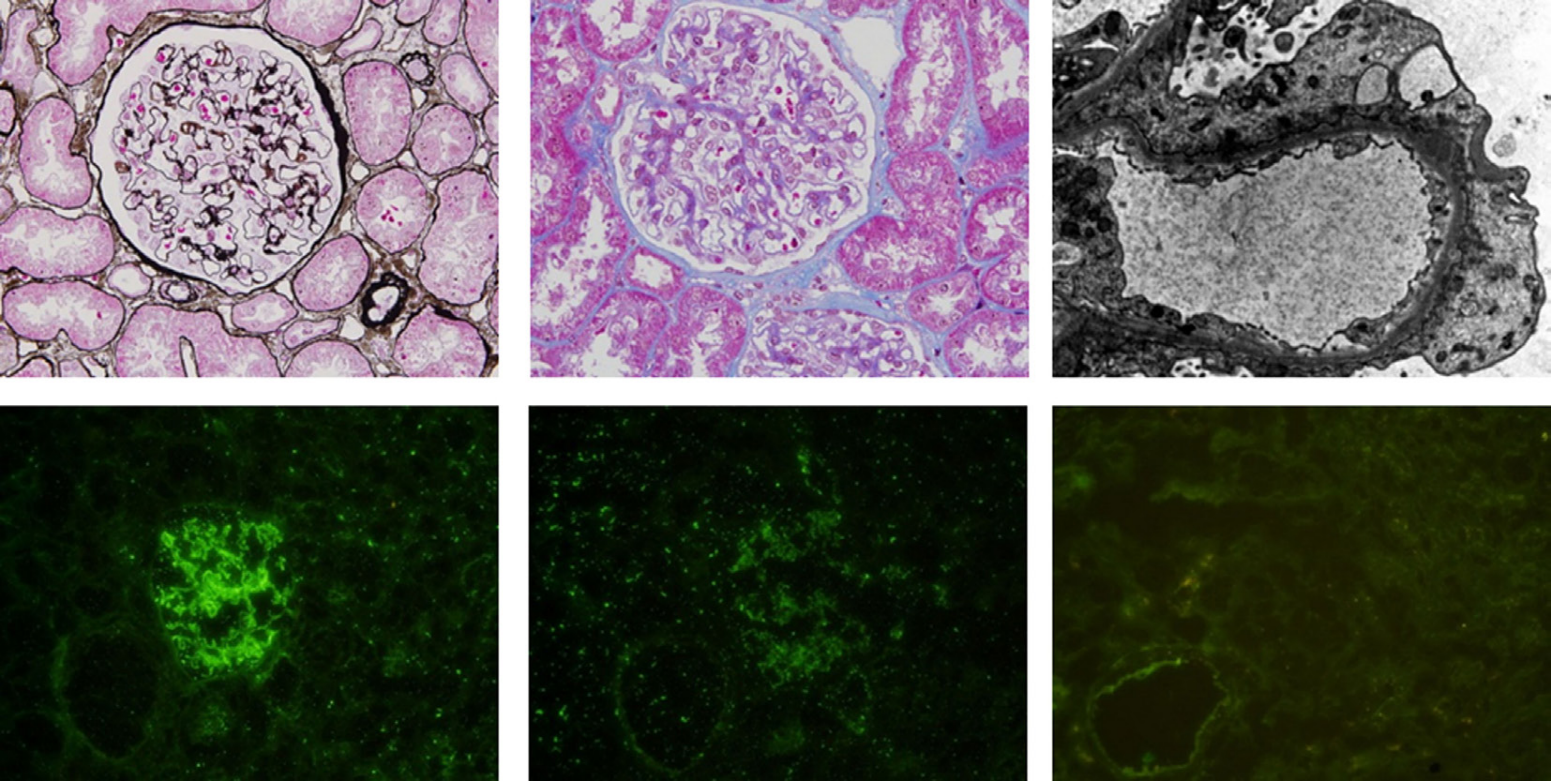
**(A)** (Above, left). Glomerular basement membrane irregular thickening and segmental spikelike projections were observed by periodic acid-silver methenamine staining. **(B)** (Above, middle). Granular fuchsinophilic protein was found to be subepithelial using Masson’s trichrome staining. **(C)** (Above, right). Electron micrograph demonstrating a few electron-dense deposits in the subepithelial basement membrane and fusions of most foot processes. Depositions of IgG **(D)** (below, left) and C3 **(E)** (below, middle) are shown by immunofluorescence microscopy. **(F)** (Below, right). PLA2R1 is negative.

A 31-year-old woman with acute monoblastic leukemia in first remission underwent HLA-identical sibling HSCT following conditioning with fludarabine and busulfan on February 24th, 2014. GVHD prophylaxis consisted of CsA and short-course methotrexate (5). Immunosuppression was tapered at +56 day posttransplantation as there was no evidence of GVHD. The patient developed lower extremity edema at 10 months posttransplantation. Laboratory analyses demonstrated a urinary protein level of 11 g/day (normal range: 0–0.14 g/day), a serum albumin level of 17.4 g/L (normal range: 40–55 g/L), and a serum cholesterol level of 10.72 mmol/L (normal range: 0–5.2 mmol/L). Immunoglobulin detection revealed low levels of IgG and normal levels of IgA and IgM. A renal biopsy specimen demonstrated glomerular basement membrane irregular thickening. Segmental spikelike projections were observed with argentaffin staining, and subepithelial granular fuchsinophilic protein was found with Masson staining under optical microscope (Figures 1A,B). An electron micrograph demonstrated a few electron-dense deposits in the subepithelial basement membrane and the fusion of most foot processes (Figure 1C). Depositions of IgG (Figure 1D) and C3+ (Figure 1E) were revealed by immunofluorescence microscopy. Additionally, PLA2R1 was negative (Figure 1F), indicating the NS was secondary. In view of the clinical presentation and histologic features, the diagnosis of membranous nephropathy was made. Based on the fact that there were no other inducements associated with acquired NS, such as history of cytomegalovirus infection, radiation, or utilization of new medicine, the NS she presented was considered a manifestation of cGVHD. No autoantibodies were found in the serum, and no other manifestations of cGVHD were observed in the patient (data not shown).

**Figure 2:**
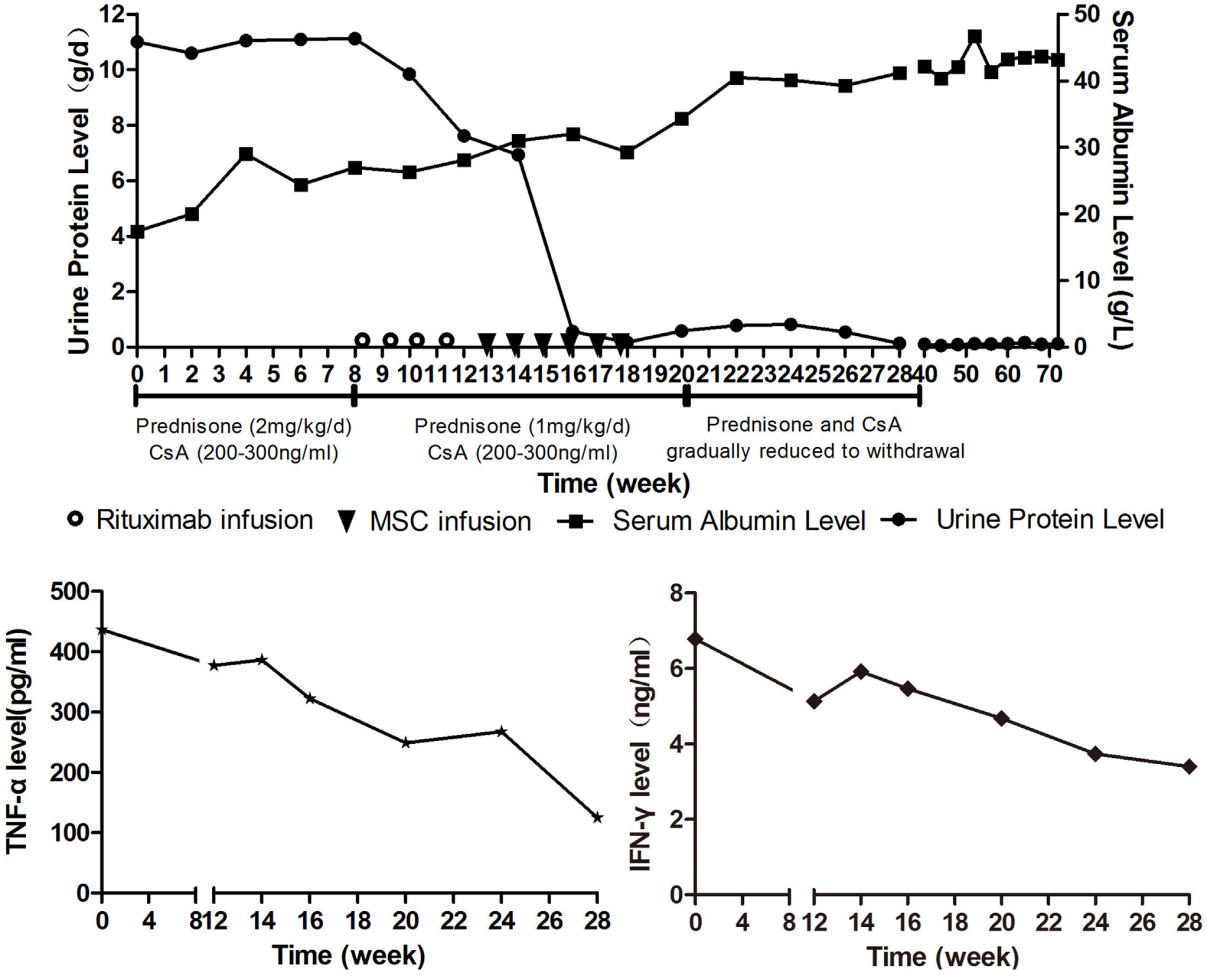
**(A)** (Above) course of proteinuria and serum albumin during treatments. Prednisone (2 mg/kg/day) and cyclosporine A (serum values maintained at 200-300ng/ml) was administrated to the patient for 8 weeks after Nephrotic syndrome diagnosis, but the urine protein remained at a high level. Rituximab was given intravenously at a dose of 100 mg weekly on day 1 of weeks 9, 10, 11, and 12. Mesenchymal stem cells (MSCs) from the bone marrow of a third-party donor were administered from weeks 13 to 18. The dose was 1 × 10^6^ cells/kg/infusion with a total of six doses planned in weekly intervals. **(B)** (Below) TNF-α and IFN-γ serum levels during treatments.

The patient was initially treated with prednisone (2 mg/kg/day) and CsA (serum values maintained at 200–300 ng/mL). Human serum albumin was administrated to the patient when her albumin level was lower than 20 g/L or edema became severe. Therapeutic effect was assessed every 2 weeks by measuring 24-h urinary protein and serum albumin levels. Criteria of therapeutic effectiveness are as follows: 1. Complete remission: normalization of serum albumin and 24-h protein levels; 2. Partial remission: increase in serum albumin level by 50% of the initial level and decrease in 24-h protein level by 50% of the initial level; 3. Stable: increase in serum albumin level by less than 50% of the initial level and decrease in 24-h protein by less than 50% of the initial level; 4. No remission: no change or deterioration of serum albumin and 24-h protein levels after treatment (3). The 24-h urinary protein level remained high after 8 weeks treatment, so the patient was considered as steroid resistant. As a consequence, rituximab was added (100 mg/week for 4 weeks) in addition to CsA and prednisone (1 mg/kg/day). Rituximab had played a certain curative effect, the patient’s urine protein level began to decline. By the time 4 weeks after the start of rituximab treatmemt, she responded with Stable as 24-h protein decreased by 31.5% (from 11.12 to 7.62 g/d). As the patient still presented with bilateral lower extremity edema and needed extra human serum albumin infusion to gain a serum albumin higher than 25 g/L, we considered the response as unsatisfactory. 11 days after the last rituximab infusion, MSCs from the bone marrow of a third-party donor were administered. The MSC dose was 1 × 10^6^ cells/kg/infusion with a total of six doses planned in weekly intervals. The patient had a response including a rapid fall in urinary protein and improvement in edema 2 weeks after the first MSC infusion. The 24-h urinary protein level and serum albumin level became normal at the sixth and 10th week after the first MSC infusion (Figure 2A). CsA and prednisone were tapered at the second week after the urinary protein was normal and withdrawn at the third month. At the time of this report, the patient has been off medication for 18 months, and no relapse of leukemia or NS has been documented.

## Discussion

As immunomodulatory cells, MSCs can sensitively receive inflammation signals to exhibit their immune and inflammation regulation effects by indirectly secreting various cytokines or chemokines and directly interacting with peripheral immune cells, inducing the generation and proliferation of Tregs and Bregs (6). MSCs have been shown to be promising tools for the treatment of refractory cGVHD with a response rate of 73.6–86.9% (6–8). Based on NS is a manifestation of cGVHD, MSCs were administered to this patient who failed first- and second-line therapy, and she eventually obtained CR. It has been reported that MSCs may ameliorate disease in rats with minimal change NS (9) and reduce acute rejection in patients who undergo renal transplantation (10). To our knowledge, this is the first report of MSCs as a treatment for NS in clinical practice.

The pathogenesis of NS after allo-HSCT remains unclear. As a manifestation of cGVHD, NS should have the same pathogenesis as cGVHD, and to some extent it does. Our previous study has indicated that NS post-HSCT is an immune disorder involving immune complex deposition, B cell, Tregs, and Th1 cytokines such as tumor necrosis factor-α (TNF-α) and interferon-γ (IFN-γ) (3, 11). A growing body of experimental and clinical evidence suggests that, except for T cells, aberrant B cell homeostasis is associated with cGVHD (12–15), and decreased Breg frequencies are associated with cGVHD severity (16). Our study demonstrated that MSC might exert therapeutic effects in cGVHD patients by modulating plasma B cell activating factor (BAFF) levels and restoring B cell homeostasis, including the promotion of CD5+ Breg (7, 17).

**Figure 3:**
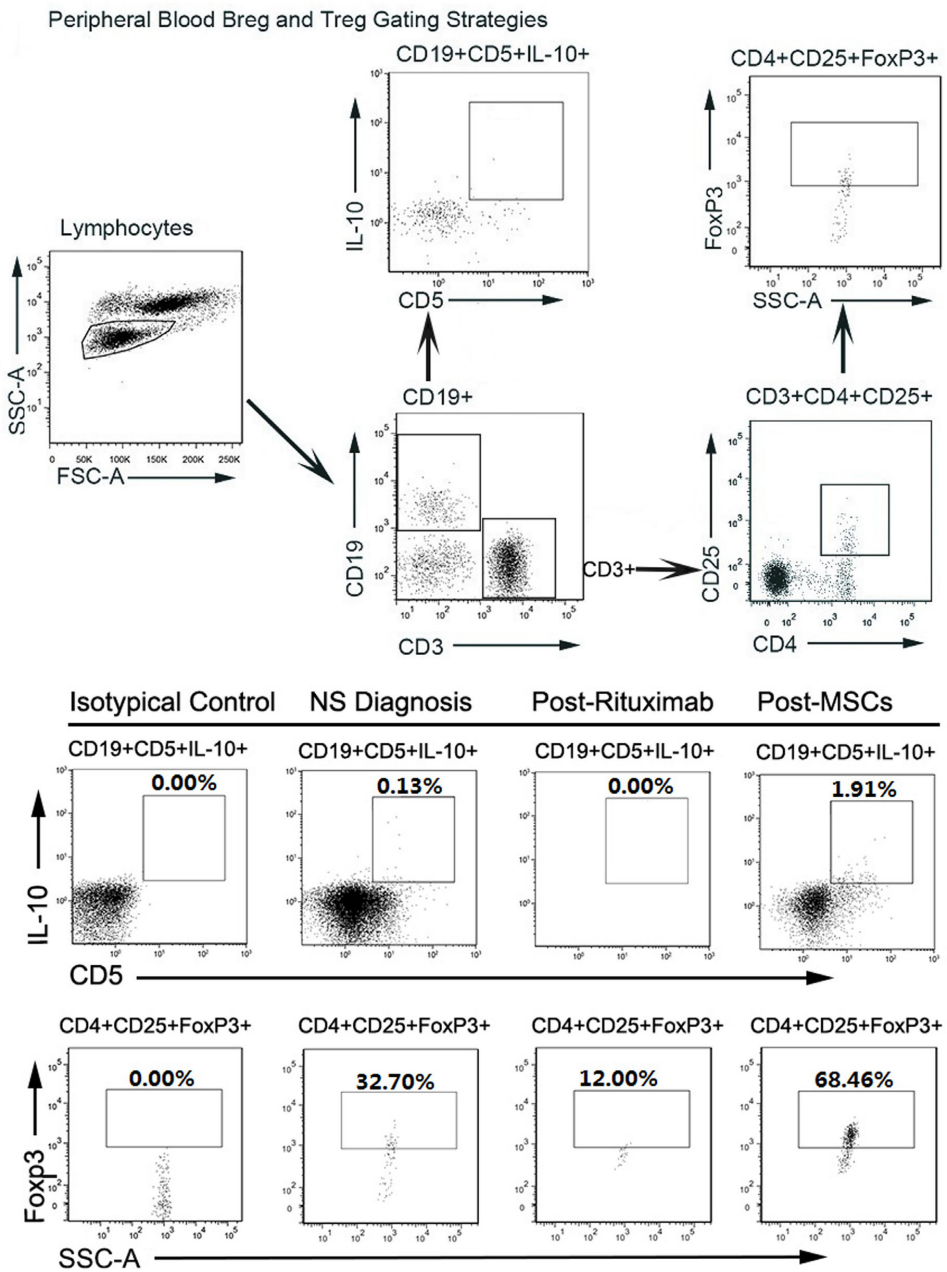
**(A)** (Above). FACS gating strategy to detect Breg and regulatory T cells (Tregs) in peripheral blood. Circulating regulatory B cells (Bregs) and Tregs were identified from the circulating lymphocyte population as CD19+CD5+IL-10+ and CD4+CD25+FoxP3+, respectively, using the gating strategy shown. **(B)** (Below). FACS results of circulating Bregs and Tregs from Nephrotic syndrome diagnosis, post-rituximab treatment, and post-mesenchymal stem cell (MSC) treatment. FSC-A, forward scatter area; SSC-A, side-scatter area.

For this patient, we analyzed peripheral B cell homeostasis, T cell subsets and TNF-α, and IFN-γ serum levels before and after treatments. The serum levels of TNF-α and IFN-γ showed a downward trend throughout the treatment process, (Figure 2B) and the Breg and Treg levels were on the rise after MSCs treatment (Figure S1 in Supplementary Material). Here, we compare the peripheral Breg and Treg level, TNF-α, and IFN-γ serum level at NS diagnosis, 4 weeks post-rituximab treatment, and 16 weeks post-MSC treatment. The proportion of CD19+ B cell was 19.84, 0.00, and 9.66% (data not shown), respectively, for NS diagnosis, 4 weeks post-rituximab treatment, and 16 weeks post-MSC treatment, and the frequency of CD5+IL-10+Breg within the CD19+ B cell population was 0.13, 0.00, and 1.91%, respectively, for these same time points. The proportion of CD4+CD25+FoxP3+ Treg among the CD4+CD25+ T cell population was 32.70, 12.00, and 68.46%, respectively, for the time points. The TNF-α serum level at these time points was 436.72, 386.65, and 124.90 pg/mL, respectively, and it was 6.78, 5.92, and 3.40 ng/mL, respectively, for IFN-γ. These results indicate that rituximab-depleted B cell, but there were no good therapeutic effects. The clinical improvement of this patient was accompanied by a decreased B cell population together with an increased frequency of Bregs and Tregs after MSC treatment (Figure 3). The immune modulatory function of Breg has recently gained increasing attention. It has been considered to be central to the maintenance of immune tolerance (18). The limited effectiveness of rituximab might be due to counterbalancing the beneficial effects of effector B cell depletion by concomitant depletion of Bregs, which was already decreased under basal conditions in this patient (18).

## Concluding Remarks

**Figure 4:**
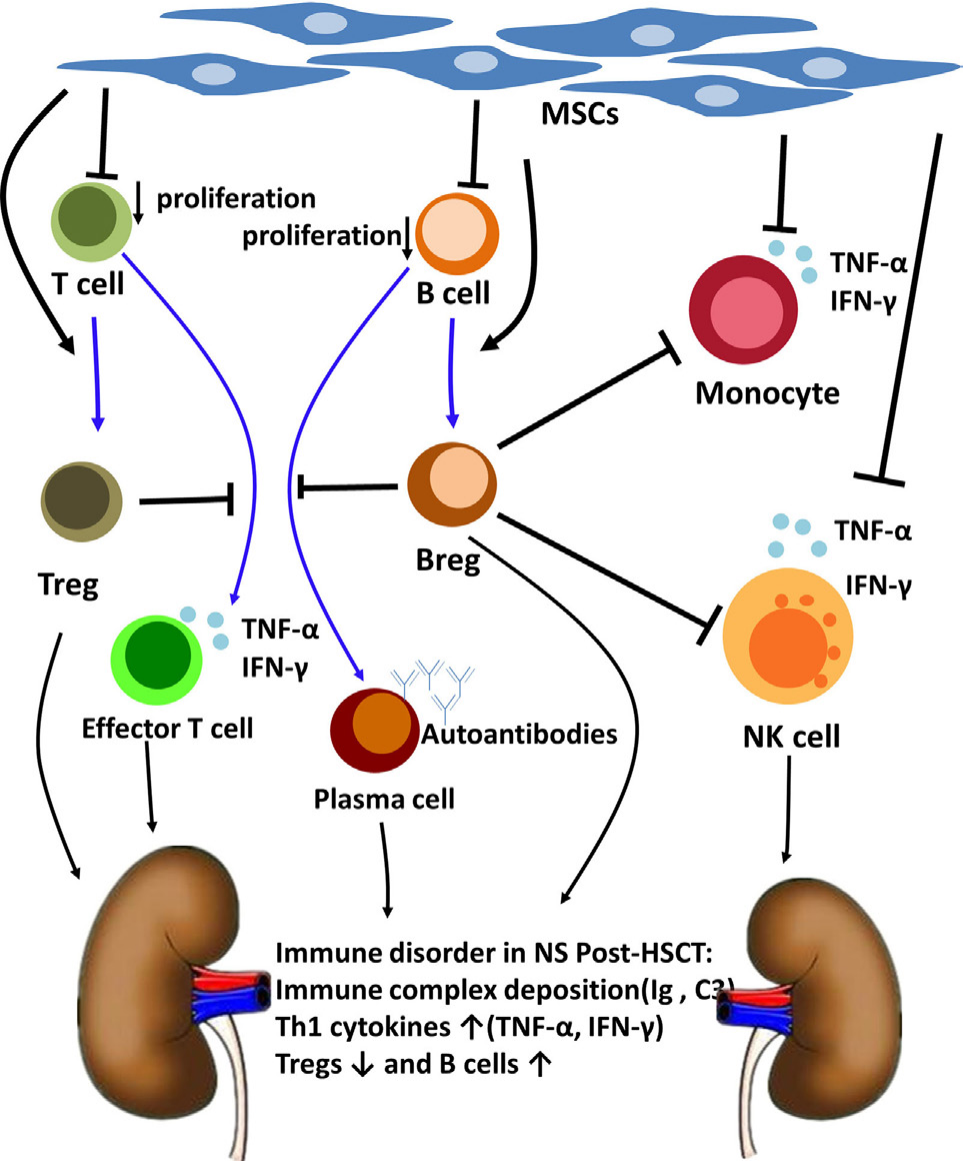
The Nephrotic syndrome (NS) immune disorder after allogeneic hematopoietic stem cell transplantation (allo-HSCT) and the immunoregulatory mechanism of mesenchymal stem cells (MSCs) and regulatory B cells (Bregs). NS post-HSCT is an immune disorder involves immune complex deposition, B cells, regulatory T cells (Tregs) and Th1 cytokines. MSCs can modulate NS after allo-HSCT by suppressing B cell proliferation, inducting Tregs and Bregs, and inhibiting inflammatory cytokines production by monocytes and NK cells. Breg cells have been considered to be central to the maintenance of immune tolerance. Bregs suppress effector T cell differentiation, support Tregs differentiation, and suppress TNF-α production by monocytes and NK cells. Bregs also suppress B cells maturation into antibody-producing plasma cells.

Based on what we have mentioned above, we speculated that MSCs could modulate NS after allo-HSCT by suppressing B cell proliferation, inducing Bregs and Tregs, and inhibiting inflammatory cytokine production by monocytes and NK cells (19–21). Among all these, Bregs might play an important role in ameliorating the NS of this patient (Figure 4). This finding might provide insight into the pathogenesis and treatment of NS after allo-HSCT and other immune-mediated NSs.

The patient has given her written, informed consent to publish the information appearing in the article.

## Author Contributions

QL, XZ, and YP have designed the paper; ZF, KZ, RL, and JS have been part of every step in this patients’ complicated diagnostic and therapeutic course and gave valuable interpretation of data. XC has done the flow cytometry analysis. All the coauthors revised paper critically and gave final approval of this version for publishing. They have ensured that all aspects of the work are accurate and have been appropriately investigated and resolved. GW, AX were their consultants (nephrologist and stem cell expert).

## Conflict of Interest Statement

The authors declare that the research was conducted in the absence of any commercial or financial relationships that could be construed as a potential conflict of interest.
