# Supplementary figures and images for: Mesenchymal Stem Cells May Ameliorate Nephrotic Syndrome Post-Allogeneic Hematopoietic Stem Cell Transplantation-Case Report
Source: Front Immunol. 2017 Aug 14;8:962. doi: 10.3389/fimmu.2017.00962 (PMC5557730; doi:10.3389/fimmu.2017.00962)

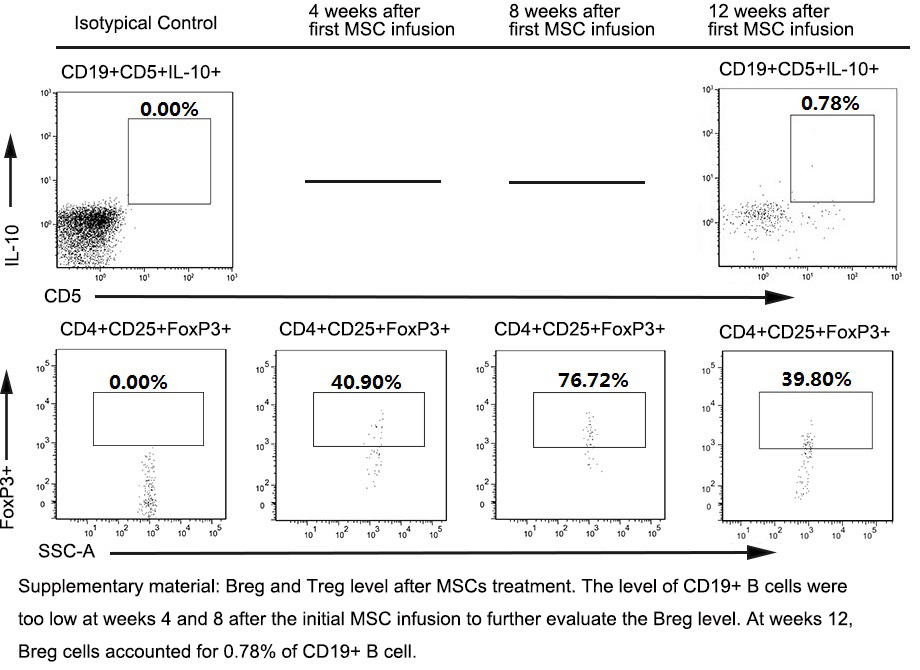

Supplement: Supplementary file 1 [file image_1.jpeg]
